# Chronological and Biological Aging in Amyotrophic Lateral Sclerosis and the Potential of Senolytic Therapies

**DOI:** 10.3390/cells13110928

**Published:** 2024-05-28

**Authors:** Anna Roshani Dashtmian, Fereshteh B. Darvishi, William David Arnold

**Affiliations:** 1 NextGen Precision Health, University of Missouri, Columbia, MO 65211, USA; aroshanidashtmian@missouri.edu (A.R.D.); fereshtehbabaeidarvishi@missouri.edu (F.B.D.); 2 NextGen Precision Health, Department of Physical Medicine and Rehabilitation, University of Missouri, Columbia, MO 65211, USA

**Keywords:** aging, sarcopenia, senescence, motor neuron

## Abstract

Amyotrophic Lateral Sclerosis (ALS) is a group of sporadic and genetic neurodegenerative disorders that result in losses of upper and lower motor neurons. Treatment of ALS is limited, and survival is 2–5 years after disease onset. While ALS can occur in younger individuals, the risk significantly increases with advancing age. Notably, both sporadic and genetic forms of ALS share pathophysiological features overlapping hallmarks of aging including genome instability/DNA damage, mitochondrial dysfunction, inflammation, proteostasis, and cellular senescence. This review explores chronological and biological aging in the context of ALS onset and progression. Age-related muscle weakness and motor unit loss mirror aspects of ALS pathology and coincide with peak ALS incidence, suggesting a potential link between aging and disease development. Hallmarks of biological aging, including DNA damage, mitochondrial dysfunction, and cellular senescence, are implicated in both aging and ALS, offering insights into shared mechanisms underlying disease pathogenesis. Furthermore, senescence-associated secretory phenotype and senolytic treatments emerge as promising avenues for ALS intervention, with the potential to mitigate neuroinflammation and modify disease progression.

## 1. Introduction

In the 1800s, Jean-Martin Charcot coined the term “Amyotrophic Lateral Sclerosis” (ALS), highlighting muscular atrophy and hardening of lateral spinal cord tissues [1]. ALS is a progressive and heterogeneous neurodegenerative disorder associated with degeneration of cortical or “upper” and spinal or “lower” motor neurons. Signs and symptoms of ALS include features of muscle weakness and wasting as well as other features of upper motor neuron dysfunction including increased tone and spasticity [2]. Progression of ALS is often rapid, with an average survival of 2–5 years after onset [3], yet progression and prognosis vary by clinical presentation, such as bulbar onset, which denotes a more poor prognosis, as well as other factors including genetics [2,4]. 

Historically, approximately 90% of ALS cases have been classified as sporadic (sALS), characterized by no family history or known genetic cause, with the remaining 10% of cases being familial ALS (fALS), associated with a positive family history or known genetic cause (fALS). Yet, as molecular testing is more readily available, it has become apparent that a significant proportion of “sporadic” ALS is associated with known mutations [5]. Comprehensive genomic analyses, including genome-wide association study and next-generation sequencing, have identified over 40 genes linked to ALS [6]. Genetic mutations in chromosome 9 open reading frame 72 (*C9ORF72*), superoxide dismutase 1 (*SOD1*), TAR DNA-Binding Protein (*TARDBP*), and Fused in Sarcoma (*FUS*) are the most common mutations associated with ALS pathology [7]. Mutations in these genes almost cover more than half of fALS cases and a proportion of sALS cases without apparent family history [7]. In 1993, mutations in the *SOD1* gene were the first to be associated with familial ALS, and since that time over 185 ALS-linked *SOD1* variants have been identified including D90A, A4V, H43R, L84V, G85R N86S, and G93A, and patients with different variants manifest varying features of onset and progression [7,8,9]. *SOD1* encodes an enzyme that contributes to antioxidant defense mechanisms through the generation of oxygen and hydrogen peroxide from superoxide species. A mutation in *SOD1* leads to variety of pathological dysfunction such as excessive reactive oxygen species (ROS) [10], as well as DNA damage and neuroinflammation [11]. TAR DNA-Binding Protein (*TDP43*) is a DNA-RNA binding protein that regulates gene expression, RNA processing, and DNA repair [12]. Fused in Sarcoma (*FUS*) is another RNA-binding protein and similarly contributes to gene expression and DNA repair mechanism [13]. The most common genetic mutation associated with ALS is the hexanucleotide (GGGGCC) repeat expansion in the non-coding region of *C9orf72* [14]. 

Treatment for ALS remains limited, and the mainstay of treatment for ALS is supportive care to manage the multifaceted impact of disease and is best delivered in a multidisciplinary clinic setting [15]. There are two disease modifying therapies that are clinically available for sALS including riluzole and edaravone, and a third, tofersen, is also available for *SOD1*-related forms of fALS [16,17]. Riluzole, approved in 1995, has an inhibitory effect on glutamate release and has shown significant efficacy in improved survival ranging up to 19 months [17,18]. Furthermore, riluzole has also been shown to reduce neuronal hyperexcitability via inhibition of sodium persistent inward currents [19]. Edaravone is a free radical scavenger that has a neuroprotective effect by reducing oxidative stress. This medication received FDA approval for intravenous (IV) administration in 2017. Subsequently, following a pivotal clinical trial of IV edaravone, the oral formulation for ALS treatment obtained FDA approval in 2022 [20]. Clinical studies showed that edaravone prolongs survival in patients with ALS by 27% [21,22]. Additionally, tofersen, an antisense oligonucleotide therapy for *SOD1*-related forms of ALS, was approved by the FDA in 2023. A phase III clinical trial study showed that tofersen reduces *SOD1* protein levels in both the cerebrospinal fluid (CSF) and blood [23]. Tofersen treatment led to reduction of plasma neurofilament light chain levels, an axonal degeneration marker [24]. However, despite these positive effects, the use of tofersen did not result in any improvement in the ALS clinical endpoint [24]. While multiple therapies are available, the combined effects are still modest and additional, more effective therapies are desperately needed. 

The pathophysiology of sporadic and genetic forms ALS is complex, and many environmental factors have been attributed to an increased risk of developing ALS. One of the most prominent risk factors is chronological age [25]. Onset of ALS before the age of 40 is uncommon and advanced age is associated with increased risk [26]. Yet, how chronological aging contributes to the risk of developing ALS remains undetermined. Understanding cellular mechanisms involved in disease onset and progression is necessary to develop more effective therapeutic interventions and advancing the understanding of how aging influences ALS disease onset and progression represents a potential avenue for exploration. The goal of this review is to discuss the intricate relationship between chronological and “biological” aging, emphasizing the accumulation of age-related damage and dysfunction as significant risk factors and drivers of disease onset and progression in ALS. Specifically, the evidence supporting senescence, a hallmark of aging, as a contributor to both ALS and the aging process is presented. The discussion will encompass the existing evidence supporting the connection between aging and ALS, offering insights into the prospects of targeting senescence as a focal point for future ALS treatments.

## 2. Chronological and Biological Aging in Disease and Neurodegeneration

Aging is one of the most important risk factors for chronic disease across biological systems [26]. The Centers for Disease Control and Prevention (CDC) reports that about 95% of older adults, aged 65 and above, have at least one chronic health condition, and nearly 80% have two or more [27]. Older adults constitute a disproportionate percentage of the population with various health conditions: 28.62% of new cancer cases are adults aged 75 and older [28,29], 29.2% of heart disease cases occur in adults over 65 years old [30], 29.2% of diabetic patients are adults over 65 years old [30], and 10% of adults aged 65 or older are diagnosed with dementia or Alzheimer’s Disease (AD) [31]. 

Chronological aging, characterized simply by the elapse of time, and biological aging, characterized by the accumulation of age-related changes in an organism, are intricately connected to the onset of organismal dysfunction across various systems [32]. The interplay between chronological and biological aging contributes to the “heterogeneity” of functional decline observed across biological systems and between individuals during the aging process. The heterogeneity of aging is a multifaceted phenomenon, influenced by a complex interplay of genetic and environmental factors, but the understanding of the heterogeneity of aging and factors that influence the rate of biological aging accumulation remains incomplete [33]. Preclinical data support that age-related decline, longevity, and response to aging-targeted pharmacological interventions depend on the genetic background of an organism [34]. HET3 mice, a model created by mating four congenic background strains of mice to obtain predictable genetic diversity, demonstrate more heterogeneity of aging phenotypes as compared with congenic strains of model mice, thus more closely mimicking the human aging phenotype [35]. Prior work in HET3 mice has demonstrated associations between the percentage of single-nucleotide polymorphism (SNP) contributions from HET3 background strains and age-related decline of neuromuscular function [35]. 

Genetic penetrance, a fundamental concept in genetics, refers to variation in the manifestation (such as age of onset or severity) of a genetic trait between individuals carrying a specific gene mutation. Genetic penetrance plays a crucial role in the heterogeneity observed in the aging process. A striking example is the wide variability that is observed across individuals carrying similar ALS-associated gene mutations. Even with shared mutations, individuals may experience symptoms at an earlier age, while others may remain asymptomatic throughout their lives [36]. For instance, incomplete and age-dependent penetrance of ALS disease is observed among individuals with the *C9orf72* mutation. Some carriers with the same mutation may manifest the disease phenotype in their twenties, while others remain asymptomatic until their nineties [37]. Understanding the genetic landscape, penetrance patterns, and the influence of biological age in ALS is crucial for elucidating the heterogeneity of penetrance, particularly with genetic forms of this debilitating condition, to pave the way for more targeted and personalized approaches to diagnosis and intervention.

## 3. Understanding ALS Incidence Rates in the Context of Aging and Neurological Decline

Muscle weakness stands out as one of the most evident and unavoidable consequences of aging. Sarcopenia, characterized by the pathological loss of muscle mass and strength with age, significantly contributes to the decline in function among older individuals, leading to mobility issues, falls, and a heightened risk of mortality [38]. Traditionally seen as primarily a muscle-related ailment, growing evidence indicates that age-related neurological changes also play a substantial role in sarcopenia [39,40]. 

**Figure 1:**
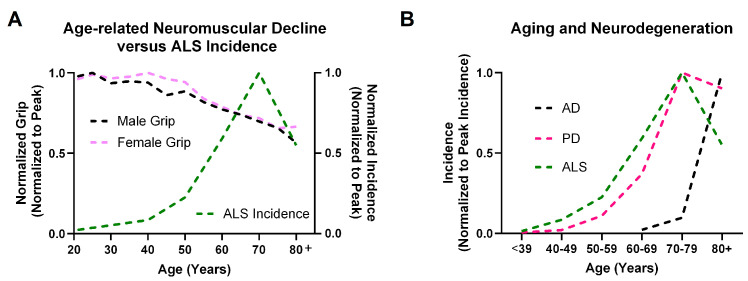
Comparison of Amyotrophic Lateral Sclerosis (ALS) with age-related decline of the neuromuscular system and with other age-related neurodegenerative disorders (Alzheimer’s Disease (AD) and Parkinson’s Disease (PD)). (**A**) Increased ALS incidence coincides with onset of grip strength decline in the general population during aging. (**B**) ALS demonstrates a divergent relationship with age as compared with AD and PD. Panel A is based on previously published data [41,42]. Panel B is based on previously published data [42,48,49].

Grip strength is a simple and common method for assessing muscle strength. Interestingly, the downward trajectory of grip strength coincides with the upward inflection of ALS incidence (Figure 1A). In Figure 1A, reference data regarding grip strength in the United States published by Wang and colleagues are compared with incidence of ALS in the United States published by Mehta and colleagues [41,42]. Grip strength remains relatively stable until mid-life (around ages 40–50) before declining, coinciding with the rapid increase in ALS incidence observed in the fifth to sixth decades of life (Figure 1A) [41,42]. 

An aspect of aging that resembles ALS involves the decline in motor neuron and motor unit number and function throughout life [43]. One of the earliest studies describing the electrophysiological method of motor unit number estimation applied this technique across various age groups and identified significant reductions in older adults [44]. Specifically, a notable decrease in the estimated number of motor units was observed after the age of 60 [44]. Consequently, the acceleration of age-related weakness and motor unit losses correlates temporally with the increasing incidence of ALS. During periods of motor unit loss, significant remodeling occurs at the neuromuscular junction, which might contribute to neuromuscular changes implicated in ALS onset and progression. Thus, the heightened occurrence of ALS is temporally linked to the age-related decline in motor units and strength. While this temporal relationship could be coincidental, mechanisms underlying age-related motor unit losses or subsequent remodeling, along with factors like disrupted muscle–nerve communication, could serve as triggers in individuals predisposed to developing ALS. In addition to spinal motor neurons, ALS results in degeneration of upper or cortical motor neurons as a distinguishing characteristic of the disease. Dysfunction of cortical motor neurons in the context of ALS is associated with clinical features of increased muscle stretch reflexes and signs and symptoms of spasticity and neurophysiological features of increased corticospinal excitability on assessments with transcranial magnetic stimulation [45]. While aging similarly results in central nervous system dysfunction including dysfunction of the motor cortex, older adults do not generally demonstrate features of spasticity and transcranial magnetic stimulation studies have generally indicated loss of excitability contrasting with ALS [46,47]. 

There are also striking differences between the incidence of ALS across ages when comparing ALS with other age-related neurodegenerative process such as AD and Parkinson’s disease (PD). The incidences of ALS, PD, and AD across ages are compared in Figure 1B based on compiled data from prior reports [48,49]. In ALS, risk prior to age of 40 is very low, peaks in after mid-life, and appears to decline in very old adults [42]. This contrasts with PD, which emerges at a similar stage in life and then incidence plateaus. Finally, AD shows later onset and progressive increase with progressing age. These data suggest that the distinct patterns of neural degeneration and clinical symptoms observed in each of these three diseases are influenced by complex interplay between aging and underlying pathophysiology. While it is important not to over interpret these patterns as these data are likely highly susceptible to artifacts such as ascertainment bias due to differences in disease natural history, rates of progression, and clinical presentation, these findings suggest that the impact of aging on ALS is complex and may differ from other age-related neurodegenerative disorders and natural aging.

## 4. Hallmarks of Biological Aging and ALS

The hallmarks of aging (HoAs), initially proposed in 2013 by Carlos López-Otín et al., constitute a comprehensive set of interconnected processes and characteristics believed to underline biological aging of organisms [50]. The original hallmarks included genomic instability, telomere attrition, altered intercellular communication, deregulated nutrient sensing, loss of proteostasis, epigenetic alterations, stem cell exhaustion, mitochondrial dysfunction, and cellular senescence [50]. These processes were described as both possible consequences and drivers of biological aging [31]. This influential framework has garnered widespread attention and has been extensively cited. Over time, subsequent reviews and discussions have further refined and expanded upon the hallmarks, reflecting the evolving understanding of aging mechanisms within the scientific community. As an age-related disorder, perhaps not unexpectedly, numerous studies have shown an association between putative hallmarks of biological aging and ALS (Table 1). The goal of this section is to provide an overview of the hallmarks of biological aging, specifically emphasizing the intersection with the HoAs including DNA damage, mitochondrial function, and senescence as shared molecular mechanisms that may contribute to the pathogenesis of both aging and ALS.

One prominent potential ALS-related mechanism that has perhaps the most obvious overlap with the HoAs involves dysregulated DNA repair, as indicated by heightened DNA damage, which has been observed in both familial and sporadic ALS cases [51,52,53]. Notably, spinal tissues from ALS patients with *C9orf72* mutations demonstrate an increase in DNA double-strand breaks and several markers of DNA damage response [54]. Telomeres are repetitive DNA sequences at chromosome ends that protect the DNA from deterioration during cell division. Progressive shortening of telomere occurs during aging and leads to an acceleration of ALS disease phenotypes. A study of a postmortem spinal cord tissue of ALS patients revealed lower telomere length [55]. The crossing of *SOD1* mice with telomerase knockout mice led to an acceleration in disease onset and lower survival rates, indicating the association of age-dependent telomere shortening with ALS penetrance [56]. Additionally, induced pluripotent stem cell-derived motor neurons (iPSCs) carrying *C9orf72* mutations exhibit an age-dependent escalation in oxidative stress and DNA damage [53]. Epigenetic alterations, encompassing changes in DNA methylation, RNA metabolism, and histone post-translational modifications, are intricately linked to ALS pathology [57]. Brain tissue from ALS patients shows increased levels of DNA methylation, including H3K9me3, H3K27me3, and H4K20me3, in comparison to healthy controls [58]. Additionally, spinal cord tissues of ALS patients display reduced microRNA levels [59]. These observations of dysregulated DNA repair mechanisms signify a distinctive feature of ALS that underscores a possible connection between DNA repair as a mechanism of biological aging and the pathogenesis of ALS. The observed elevated DNA damage, impaired DNA repair, and epigenetic alterations collectively highlight a shared molecular mechanism that may contribute to both the aging process and the development of ALS and could represent novel targets for treatment strategies. Various ALS-related genes are known to play a significant role in the DNA damage response (DDR). Within neurons, *TDP43* is involved in DDR, and its removal from the nucleus in spinal motor neurons results in defects in DNA double-strand break (DSB) repair in ALS [60]. *FUS* is another ALS-related gene that appears to play a role in DNA repair mechanisms by contributing to the assembly of damaged DNA with repair proteins [13]. A mutation in C9rof72, a major cause of familial ALS, is associated with a significant increase in DDR markers [54]. Moreover, the *SOD1* gene is engaged in DDR by regulating transcription within the nucleus [61]. Although there is a strong association between DNA damage and ALS pathology, the comprehension of DNA repair mechanisms as a potential therapeutic intervention for ALS is not fully understood. 

Several studies have highlighted the presence of mitochondrial abnormalities in ALS, another key HoA [62,63]. Examination of mitochondrial changes at various stages of ALS indicates a defect in mitochondrial axonal transport or morphology preceding the manifestation of motor symptoms in familial ALS mouse models carrying *SOD1* and *TDP43* mutations [64]. Experimental manipulations of *TDP43*, including its suppression, overexpression, or mislocalization, have been linked to abnormalities in mitochondrial dynamics and transport within primary motor neurons [65]. An in vitro model of ALS has provided evidence of fragmented mitochondria and an increased level of mitochondrial fission proteins, such as Drp1/Fis1, accompanied by elevated reactive oxygen species (ROS) levels. These findings strongly suggest the crucial involvement of mitochondrial dynamics in the progression of ALS and strongly suggest a significant overlap in the underlying mechanisms of ALS with specific HoAs [66]. 

Cellular senescence, a state characterized by irreversible cell cycle arrest, was coined in 1961 to describe the permanent loss of cellular proliferation in human somatic cells [67]. Senescence has beneficial effects during development and adulthood that include elimination of unwanted cells and tissue remodeling during mammalian embryonic development [68]. While senescence in post-mitotic cells remains less understood compared to other cell types, evidence from studies on aged mouse models and humans suggests that this phenomenon is not limited to proliferating cells and neurons may activate a regulatory mechanism and adopt a senescence-like phenotype [69]. In the adult organism, senescence serves as a potential protective mechanism, preventing uncontrolled proliferation of damaged or tumor cells [70]. However, as a HoA, senescence is also considered to have detrimental outcomes where it occurs in response to factors like telomere shortening, accumulated DNA damage, excessive reactive oxygen species (ROS) production, and dysregulated mitochondria. Detection of cell cycle arrest markers such as P16 and P21 and senescence-associated secretory phenotype (SASP) components such as interleukin-6 (IL-6) and interleukin-8 (IL-8) in neuronal or glial cell populations support an association between cellular senescence and disease progression in ALS. 

Studies in ALS patients have revealed a systemic increase in senescent cells, particularly notable in patients with bulbar involvement [71]. Muscle biopsy and cultured myoblasts from ALS patients have shown elevated senescence markers [72]. Co-immunofluorescence staining of neuronal or glial markers with general cell cycle regulatory markers like P16 and P21 demonstrated increased expression of these markers in astrocytes and neurons within the motor cortex of ALS patients [73]. Activation of senescence in these cells underscores the intricate association between senescence as a hallmark of aging and the pathogenesis of ALS. 

**Table 1:** Evidence of differential effects on hallmarks of aging in patients and models of Amyotrophic Lateral Sclerosis.

Gene	Hallmarks of Aging	Evidence in ALS	References
*C9orf72* *TDP43* *FUS* *SOD1*	Genome Instability 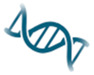	Patient, iPSC-derived motor neuron Patient, iPSC-derived motor neuron, SH-SY5Y cells Patients, iPSC-derived motor neuron, mouse model Patients, cell models and mouse models sALS patients	[53,54,74,75,76,77,78] [79,80,81,82] [83,84,85,86] [12,87,88,89,90,91,92,93] [81,93,94]
*C9orf72* *TDP43* *FUS* *SOD1*	Epigenetic Alterations 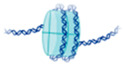	Patient, iPSC-derived motor neuron Patient, SHSY5Y cell model Patients, EPSC- and iPSC-derived motor neuron, mouse, and yeast model sALS patients	[95,96,97] [57,98,99] [100,101,102] [97,103,104,105] [15,16,106,107,108,109]
*C9orf72* *SOD1* *TDP43*	Inflammation 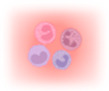	Patients, mouse, and cell model Patients, mouse, rat, and cell models Mouse model sALS patients, iPSC-derived astrocyte from sALS patients	[110,111,112,113] [66,111,113,114,115,116,117,118] [78] [119]
*SOD1* *C9orf72* *TDP-43* *FUS*	Mitochondrial Dysfunction 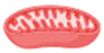	Patients, mouse, and rat model Patients, iPSC-derived motor neurons PSC-derived motor neurons, fibroblast from patients, mouse model fALS tissue, primary neuron	[62,63,64,120,121,122,123] [75,124] [64,66,75] [125,126,127]
*C9orf72* *TDP43* *FUS* *SOD1*	Loss of Proteostasis 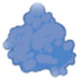	Primary neurons, NSC-34 cells, mouse model Patients, mouse model, Zebrafish, *Caenorhabditis elegans*, Neuro2 cell model Patients, mouse model Patients, mouse model sALS	[128,129,130,131] [128,131,132,133,134,135] [128,131,136] [128,131,137] [138]
*C9orf72* *SOD1*	Cellular Senescence 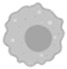	Patients, iPSC-derived astrocyte from patients Patients, mouse, and rat models Myoblast from sALS patients sALS patients	[73,113] [139] [72] [71,73]

## 5. Senescence-Associated Secretory Phenotype (SASP) and Senolytic Treatments

A subset of senescent cells enter a pathological state and undergo significant changes in their secretome, characterized by a notable abundance of pro-inflammatory cytokines, reactive oxygen species, and matrix metalloproteinases [140]. This phenomenon is known as the ‘senescence-associated secretory phenotype’ (SASP) [141,142]. SASP is recognized as a maladaptive phenotype of senescent cells which plays a pivotal role in chronic low-grade sterile inflammation, commonly referred to as “inflammaging” [143,144]. SASP contributes to progressive tissue dysfunction and accelerated senescence of surrounding tissues with repercussions extending to various cell types, including neurons and glial cells, and contributes to biological aging [145]. In ALS, this sustained inflammatory response can exacerbate neuroinflammation and contribute to the degeneration of motor neurons.

Glial cells, including microglia and astrocytes, have an intricate role in maintaining neural health. Microglia protect the central nervous system, including motor neurons, against immune challenges, while astrocytes contribute to regulation of excitability, ensuring finely tuned neural signaling and activity. However, there is growing evidence that, during aging, glial cells may acquire maladaptive SASP-related phenotypes and lose their typical supportive function [146]. Furthermore, evidence derived from brain and spinal cord tissues from both familial (*SOD1* and *C9orf72*) and sporadic ALS patients suggests that glial cell senescence may be accelerated in the context of ALS [111]. This acceleration involves the continuous glial release of pro-inflammatory cytokines and chemokines, propagating SASP to neighboring glial cells and other tissues [66,112,114]. The presence of senescent cells within affected regions of the CNS in the ALS mouse model may promote the aggregation of proteins and contribute to neuronal loss [147]. Additionally, accumulation of senescent glial cells and their SASP can contribute to excitatory and inhibitory synapse loss and neuronal degeneration [148]. Examinations of in vivo models of ALS with differing progression rates reveal a strong correlation between the inflammatory response and the progression of the disease [149]. This underscores the significance of understanding how inflammatory processes evolve in conjunction with the advancement of ALS pathology, providing valuable insights into the complex dynamics underlying the disease progression [149]. Assessing the temporal dynamics of inflammatory response in the limb muscle of ALS *SOD1* rats reveals a strong correlation between a constant increase in inflammatory markers and disease progression [114]. Notably, the crucial inflammatory chemokine, monocyte chemoattractant protein 1 (MCP1), exhibits a significant increase in motor neurons and microglia in slow-progressing ALS mouse models, correlating with disease progression [118]. The hm*SOD1* transgenic rat model demonstrates that both age and disease progression contribute to an increase in the level of CD11b in both ventral nerve roots and the sciatic nerve. Microglial aggregation in the ventral horn is observed before the onset of disease symptoms [117]. Elevated levels of inflammatory markers in astrocytes and microglia, such as GFAP and CD68, progressively elevate through disease progression, underscoring the pivotal involvement of the immune response and glial activity in shaping the ALS phenotype [114].

**Figure 2:**
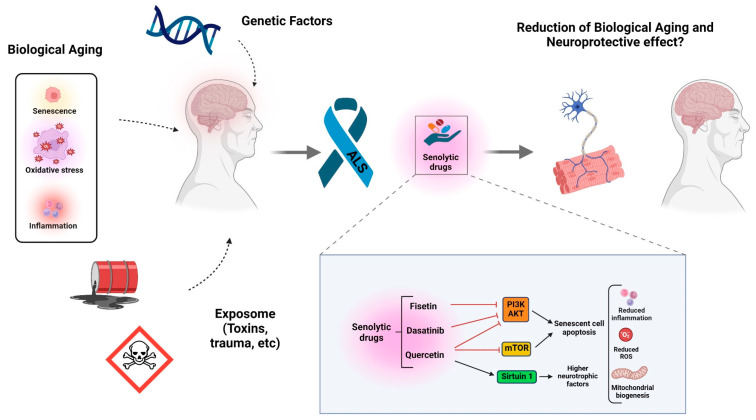
Schematic representation of aging signatures in ALS and targeting cellular senescence with senolytic agents as a potential therapeutic approach. Senolytic drugs can selectively target key proteins and apoptotic signaling molecules, effectively eliminating senescent cells and diminishing the senescence-associated secretory phenotype (SASP), along with its associated consequences. Black arrows represent activation. Red blunt-ended lines represent inhibition. PI3K: phosphoinositide 3-kinase, AKT: serine/threonine kinase Akt (also known as protein kinase B or PKB), mTOR: mammalian target of rapamycin, ROS: reactive oxygen species. (Figure created using Biorender).

The removal of senescent cells has demonstrated a positive impact in the context of aging and neurodegeneration with an improvement in age-related brain inflammation as well as cognitive and motor function in aged mice [150]. Clearance of senescence in models of AD and PD has led to a reduction in neuroinflammation and improved disease phenotypes. Senolytics, small molecules selectively targeting cells with senescence-like phenotypes, have emerged as a potential intervention strategy. These agents aim to improve cellular function and tissue health by reducing the senescent cell burden accumulated during aging (Figure 2). 

Examples such as dasatinib, quercetin, and fisetin have shown promise. Dasatinib inhibits a variety of SCR family tyrosine kinases, resulting in apoptosis of cells with senescence-like phenotypes through disinhibition of caspase 3, 7, and 9. Additionally, dasatinib regulates the cell cycle through its inhibitory effect on the pro-survival PI3K/AKT pathway [151]. In an in vitro and in vivo model of pancreatic cancer, dasatinib played a role in reducing tumor growth by affecting angiogenesis, cell motility, and migration [152,153]. Furthermore, dasatinib has been shown to promote cell death in different tumor cells [154]. As a micronutrient, quercetin and fisetin are found in our daily diet. These bioflavonoids are beneficial molecules which provide widespread health benefits through their antioxidant and anti-inflammatory activity. Additionally, quercetin and fisetin ameliorate tissue damage by targeting senescent cells through inhibition of mTOR and PI3K [152,155]. A study showed that adding quercetin to mice diets enhanced exercise tolerance by improving mitochondrial biogenesis in muscles and the brain [156]. The administration of low-dose quercetin exhibited geroprotective effects in aged wild-type mice, showing reduced hair loss, maintained normal levels of blood glucose, bone material density, and elevated exercise endurance when compared to the vehicle group [157]. In a separate investigation using senescence-accelerated mice, quercetin demonstrated the capacity to enhance learning and cognitive function [158]. The neuroprotective effect of quercetin is attributed to the inhibition of neuroinflammatory markers, including IL-1B and IL-18, a decrease in hippocampal ROS levels, and elevation in a synaptic marker (PSD95) and neurotropic factors (BDNF and NGF). The anti-inflammatory properties of quercetin were further attributed to its modulation of the longevity-associated molecule SIRT1 and inflammasome by the SIRT1/NLRP3 pathway [158]. A senolytic cocktail of dasatinib and quercetin demonstrated a reduction in the number of senescent cells and inflammatory cytokines and improvements in physical function and survival rate in aged mice [159,160]. Fisetin has been shown to improve brain function by antagonizing proinflammatory responses in age-related disorders [155]. Treating aged mice with fisetin resulted in lower levels of senescent cells, ROS, and inflammation, as well as extending the life span of the aged animals [155]. The antioxidant activity and reduction in ROS levels by current ALS treatments such as riluzole and edaravone indicate that there might be a shared molecular mechanism between current ALS treatments and senolytic therapies [161,162]. Recognizing these overlapping pathways could pave the way for potential synergistic effects or therapeutic benefits when combining these treatments.

In the context of neurodegenerative diseases, including ALS, senolytic drugs hold promise in mitigating neuroinflammation and reducing pro-inflammatory molecules in the nervous system. A recent phase 1 feasibility trial of senolytics on AD patients demonstrated a significant reduction in the accumulation of cortical neurofibrillary tangles, associated with decreased brain cortex atrophy and the restoration of normal blood flow in the cerebrum [163]. The substantial evidence linking senescence and its associated phenotype to ALS suggests that it can exacerbate symptoms such as muscle weakness, fatigue, pain, and cognitive impairment in ALS patients. By reducing senescent cell burden, senolytic therapies may alleviate these symptoms and improve overall comfort and well-being. ALS is characterized by progressive loss of motor function, leading to increasing dependence on caregivers for activities of daily living. Senolytic therapies that preserve muscle strength and motor function could enhance patients’ ability to perform daily tasks independently, thereby maintaining a higher level of functional autonomy. 

While preclinical research has demonstrated promising outcomes, translating these findings into clinical applications necessitates rigorous investigation. Conducting dedicated clinical trials to assess the effectiveness and safety of senolytic drugs in individuals with ALS is crucial. Additionally, understanding the specific mechanisms by which senescence contributes to ALS pathology and how its elimination influences disease progression is paramount for developing targeted and effective therapeutic strategies. The potential of senolytics in ALS offers a novel avenue for therapeutic intervention that warrants comprehensive exploration and further investigation.

Integrating lifestyle modifications alongside conventional treatments could offer a more holistic approach to disease management and improve overall quality of life for ALS patients. Dietary habits can influence various biological processes implicated in aging and ALS pathogenesis [164]. For instance, a diet rich in antioxidants, bioflavonoids, and phytochemicals found in fruits, vegetables, and fish may possess neuroprotective properties and help mitigate oxidative stress and inflammation associated with both aging and ALS [165]. Conversely, diets high in saturated fats and processed foods may exacerbate inflammatory responses and accelerate disease progression [166]. Exercise promotes neurogenesis, enhances mitochondrial function, and stimulates the production of neurotrophic factors, which could potentially slow down neurodegeneration in ALS [167]. Additionally, exercise can improve cardiovascular health, maintain muscle strength, and alleviate symptoms such as muscle cramps and fatigue commonly experienced by ALS patients [168]. Chronic stress can accelerate cellular aging processes and exacerbate neuroinflammation, which may worsen ALS symptoms [169]. Exposure to environmental toxins and pollutants has been implicated in neurodegenerative diseases, including ALS. Minimizing exposure to heavy metals, pesticides, and air pollutants through lifestyle modifications and environmental interventions may help reduce oxidative stress and neuroinflammation, thus potentially slowing down ALS progression [170]. By considering these lifestyle factors and implementing targeted interventions, individuals living with ALS may have the opportunity to positively influence biological aging processes and potentially attenuate disease progression.

Many senolytic compounds share the same targets and may have potential off-target effects. Identifying senolytic compounds with a favorable safety profile is crucial for their clinical development. Developing senolytic therapies that effectively target the specific senescent cell subpopulations implicated in ALS pathogenesis is crucial for therapeutic success. Designing well-controlled clinical trials with appropriate patient selection criteria, outcome measures, and treatment regimens is essential for assessing the safety and efficacy of senolytic therapies in ALS. Longitudinal studies with large patient cohorts are needed to evaluate the long-term effects of these therapies on disease progression and patient outcomes. Addressing these limitations requires interdisciplinary collaboration among researchers, clinicians, and pharmaceutical companies to advance the development of senolytic therapies for ALS while ensuring patient safety and therapeutic efficacy.

## 6. Conclusions

The pathophysiological mechanisms of ALS remain incompletely understood. A wide range of genetic variants are attributed to an increased risk of ALS, but in many of these, age is a major factor in the development of ALS. This review highlights the connection between aging and ALS, emphasizing the overlap of senescence and SASP in the disease’s development. Although elimination of senescence using senolytic drugs has shown positive outcomes in aging and other neurodegenerative disorders, application of senolytics in the context of ALS remains to be fully explored. Further research targeting the accumulation of biological aging factors may be a viable option for treating and preventing the onset of ALS, particularly in individuals with presymptomatically identified ALS gene risk variants.
